# Long-Term Water Stability Analysis of Graphene-Composite-Modified Permeable Asphalt Mixture

**DOI:** 10.3390/ma18215024

**Published:** 2025-11-04

**Authors:** Suzhan Ji, Yu Li, Xu Wu, Ke Liang, Xiaojian Cao, Xiaoguang Yuan, Qiangru Shen

**Affiliations:** 1 School of Transportation and Civil Engineering, Nantong University, Nantong 226019, China; 2333320028@stmail.ntu.edu.cn (S.J.);; 2 China Communications Construction Corporation General Contracting and Operation Branch, Beijing 100088, China; 3 Institute of Smart O&M and IoT, Nantong Institute of Technology, Nantong 226001, China; 4 Nantong Construction Engineering Quality Testing Center, Nantong 226000, China

**Keywords:** permeable asphalt mixture, graphene, multi-factor coupled rutting, energy dispersive spectrometer, RGB algorithm

## Abstract

To investigate the long-term water stability of graphene-modified permeable asphalt mixtures, in this study, we analysed the effects of single factors and multi-factor coupling. The single-factor water stability was investigated through the free thawing splitting test, standard Cantabro test, and immersion Cantabro test; the experimental indicators were the freeze–thaw cracking ratio (TSR), mass loss rate, and immersion mass loss rate, respectively. The multi-factor water stability was studied through immersion operation tests of mixtures with different degrees of ageing. The dispersion of graphene was examined through Raman mapping, the formation of three-dimensional network structures of graphene and SBS was evaluated via the dynamic shear rheometer test (DSR), and the elemental distribution was quantitatively analysed using energy-dispersive spectroscopy (EDS) and an image pixel algorithm (RGB). The results indicate that an unaged graphene-composite- and SBS-modified permeable asphalt mixture with an optimal graphene content of 0.05% demonstrated a 4.5% improvement in the TSR, alongside reductions in the mass loss rate and water immersion mass loss rate of 25.64% and 23.52%, respectively. Even after prolonged thermal oxygen ageing, its TSR, mass loss rate, and water immersion mass loss rate improved by 5.1%, 23.04%, and 20.70%, respectively. Multi-factor coupling tests confirmed that the water stability met requirements under severe conditions, with better performance at high temperatures. Graphene was uniformly dispersed in the modified asphalt. The appearance of a plateau region at low frequencies in graphene-composite- and SBS-modified asphalt verified the formation of a three-dimensional network structure, and the oxygen content was positively correlated with deepening thermal oxidative ageing.

## 1. Introduction

To achieve sustainable urban development, permeable asphalt mixtures serve as pavement materials for sponge cities [1] due to their excellent noise reduction, drainage, and anti-slip properties [2,3,4,5]. Permeable asphalt mixtures possess a distinctive high porosity structure (18–25%), an open-graded design preparation method, and a composition featuring highly viscoelastic composite-modified bitumen, distinguishing them from dense-graded asphalt mixtures.

With the advancement of nanotechnology, nanomaterials such as graphene exhibit outstanding electrical conductivity, mechanical properties, and physical characteristics [6,7,8]. Additionally, they demonstrate exceptional resistance to ageing [9,10]. Graphene, as a novel compound, finds extensive application in civil engineering materials [11,12,13,14]. Asphalt mixtures produced using graphene modifiers have emerged as a current research focus. Li, Ruixia et al. [15] analysed the combined effects of ageing and salt–alkali coupling on the fatigue and self-healing properties of asphalt materials by comparing 70-grade base asphalt with graphene oxide-modified asphalt; Adnan, Abbas Mukhtar et al. [16] investigated the effects of graphene modifiers on the compatibility and rheological behaviour of tire-rubber-powder-modified asphalt through rheological experiments; the results indicate that graphene incorporation enhances modified asphalt’s resistance to rutting, ageing, and fatigue. Zhao, Yian et al. [17] investigated the grey correlation between graphene oxide framework (GOF) content and pavement performance through rutting tests, water immersion Marshall tests, three-point bending beam tests, and freeze–thaw splitting tests. The results indicate a positive correlation between GOF content and the pavement performance of asphalt mixtures. Yong, Peng et al. [18] conducted a series of tests including rutting, Kenta-Borg scattering, and freeze–thaw splitting. The results indicate that GO composite-rubber powder–asphalt mixture (prepared using 70# asphalt as the base binder, modified with 0.4 g of GO and 40-mesh rubber powder; coarse aggregate: limestone gravel; fine aggregate: manufactured sand; mineral powder: limestone powder; SMA-13 grading type) exhibited the most outstanding rutting resistance and water stability.

Permeable asphalt mixtures are more susceptible to water erosion [19], leading to pavement deterioration such as high-speed surface spalling, cracking, and fragmentation [20]. This reduces the cohesion between the asphalt and aggregates [21] and ultimately diminishes the water stability of the mixtures. Additionally, heavy traffic loads and ageing effects during paving and the service life of permeable asphalt mixtures further diminish water stability performance [22,23]. As of now, various scholars have researched factors affecting the water stability of asphalt mixtures. Shoaib Md Shahnewaz et al. [24] tested the water stability of bamboo fibre permeable asphalt mixtures with concentrations of 0.2%, 0.3%, 0.4%, and 0.5% through the Cantabro and permeability tests. Valerio Carlos Andrés-Valer et al. [25] used cellulose ash (CA) and combustion soot (CS) as regenerated fillers, and the water stability of permeable asphalt mixtures prepared from the regenerated fillers was tested through the Cantabro, freeze–thaw splitting, and Marshall tests. Xiao, Yue et al. [26] conducted comparative experiments to investigate the effects of lignin fibre, polyester fibre, and polypropylene fibre modifiers on the water stability of permeable asphalt mixtures under static and dynamic water conditions. The results indicated that polyester fibre modifiers exhibited the best water stability performance.

In the process of microscopic mechanism analysis, the distribution laws of surface elements in permeable asphalt mixtures under different ageing and water immersion conditions were analysed using energy spectra [27]. The dispersion of graphene was investigated through specific 2D bands in Raman tests [28]. Furthermore, Yadykova, AY et al. [29] found that in the dynamic shear rheometer (DSR) test, the modified asphalt G′ changed little within the low-frequency range, and there was a plateau region. Rheological analysis indicated the formation of a three-dimensional network structure.

Graphene has high water stability when used in the preparation of permeable asphalt mixtures. However, at present, the relatively high cost of graphene limits its wide application. In the long run, with the advancement of technology, the production cost of graphene-modified permeable asphalt mixtures will become feasible in practical engineering.

In conclusion, scholars have conducted single-factor studies on the water stability of permeable asphalt mixtures under the influence of temperature, ageing conditions, and large traffic loads. However, there is a lack of systematic and in-depth research on the water stability performance of such compounds under the coupling of multiple factors during long-term use. Based on this, in this study, we innovatively investigated both unaged and aged graphene-modified permeable asphalt mixtures. Single-factor analysis was adopted to investigate the water stability of these mixtures under freeze–thawfreeze-thaw cycles, loads, and hydrothermal action. In addition, the influence laws of multiple factors —temperature, ageing degree, and heavy traffic load—on the water stability were also evaluated. Meanwhile, the Raman mapping test was used to verify the uniformity of graphene dispersion, and the dynamic shear rheometer test in a low frequency range was used to analyse the formation of the three-dimensional network structure of the graphene-composite- and SBS-modified asphalt. Energy-dispersive spectroscopy and the RGB image algorithm were used to quantitatively analyse the elemental distribution.

## 2. Test Materials and Methods

### 2.1. Test Materials

This study employed 70# base asphalt as the foundation material for preparing styrene-butadiene rubber (SBS)-modified asphalt and graphene-composite- and SBS-modified asphalt. The graphene’s primary technical specifications are outlined in Table 1. The molecular weight of the SBS material was 100,000, with styrene accounting for 30% by weight. Basalt was selected for both coarse and fine aggregates, while limestone served as the mineral filler. Basalt, the most widely used aggregate in road pavements, is characterised by its dense, hard structure and high abrasion resistance. It effectively reduces water ingress and exhibits excellent adhesion to asphalt [30]. As an alkaline aggregate, limestone enhances the water stability of permeable asphalt mixtures through its surface morphology and chemical composition [31].

### 2.2. Preparation of Modified Bitumen

**Figure 1:**
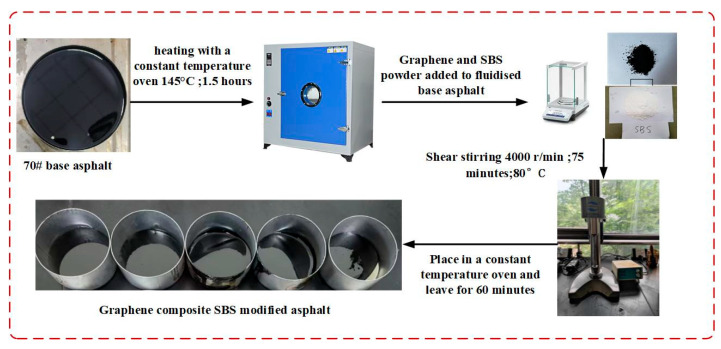
Preparation of graphene-composite and SBS-modified asphalt.

This study employed SBS at a 7.5% dosage for the preparation of modified bitumen. At this dosage, bitumen exhibits favourable softening point, ductility, and ageing resistance properties [32]. This was blended with 0.05%, 0.1%, 0.15%, or 0.2% graphene to prepare graphene-composite-modified permeable asphalt mixtures as experimental groups. A mixture prepared from 7.5% SBS-modified bitumen without graphene addition served as the control group. During the preparation of bitumen, DMSO solvent—selected for its high polarity and intercalation capacity with graphene layers—was employed to pre-treat the graphene before its incorporation into the bitumen system [33,34]. In the pre-treatment, graphene and DMSO solvent were mixed at a mass ratio of 1:100 and subjected to centrifugation at 8000 rpm for 20 min. Following this, the mixture was dried in an 80 °C vacuum oven for 12 h to completely remove the DMSO solvent solution. The preparation process of graphene is shown in Figure 1.

### 2.3. Graphene-Composite-Modified Permeable Asphalt Mixture Mix Design

**Figure 2:**
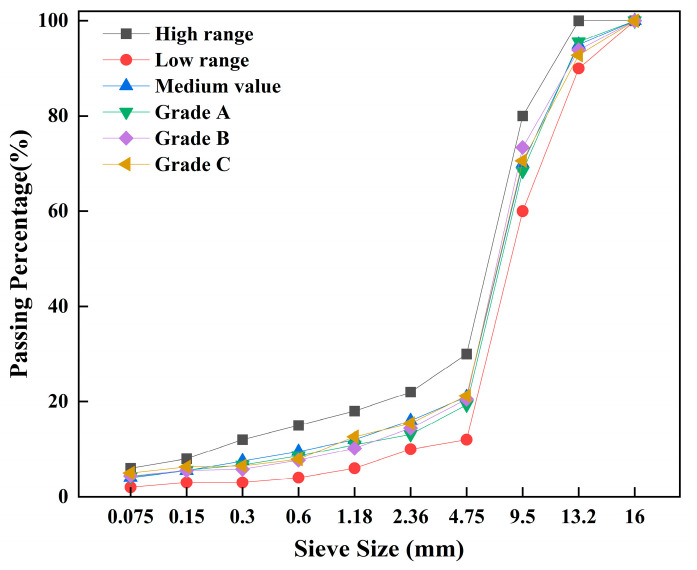
Grading of porous asphalt mixtures.

The aggregate grading for the graphene-modified permeable asphalt mixture was designed in accordance with ‘the provisions of the Technical Specification for Construction of Highway Asphalt Pavements (JTG F40-2004)’ [35]. Equations (1)–(5) are derived from the JTG F40-2004 specification. We designed three distinct aggregate gradations, A, B, and C, with the respective gradation profiles shown in Figure 2. Taking the asphalt film thickness h = 14 μm, calculations using Equations (1) and (2) yield asphalt–aggregate ratios a_1_, a_2_, and a_3_ for A, B, and C of 4.91%, 4.87%, and 5.42%, respectively. Calculations using Equations (3)–(5) indicate that 4.91% represents the optimum asphalt–aggregate ratio.

P_b_ = h × A (1)

(2) $$A=(2+0.02a+0.04b+0.08c+0.14d+0.3e+0.6f+1.6g)/48.74(m^{2}/kg)$$

where $P_{b}$ is the asphalt-to-aggregate ratio, A is the total surface area of the aggregate, and a, b, c, d, e, f, g are the pass percentages for the 4.75, 2.36, 1.18, 0.6, 0.3, 0.15, and 0.075 mm sieve apertures, respectively.apertures respectively.

(3) $${\mathrm{OAC}}_{1}=(a_{1}{+a}_{2}{+a}_{3})/3$$

(4) $$OAC_{2}=\frac{OAC_{min}+OAC_{max}}{2}$$

(5) $$OAC=\frac{OAC_{1}+OAC_{2}}{2}$$

In the formulas, a_1_, a_2_, a_3_ denote the oil-to-aggregate ratios for gradations A, B, and C, respectively; OAC_min_ and OAC_max_ represent the minimum and maximum asphalt–aggregate ratios, respectively; and OAC and OAC_1_ denote the optimum asphalt–aggregate ratio and initial asphalt–aggregate ratios, respectively.

### 2.4. Test Methods

**Figure 3:**
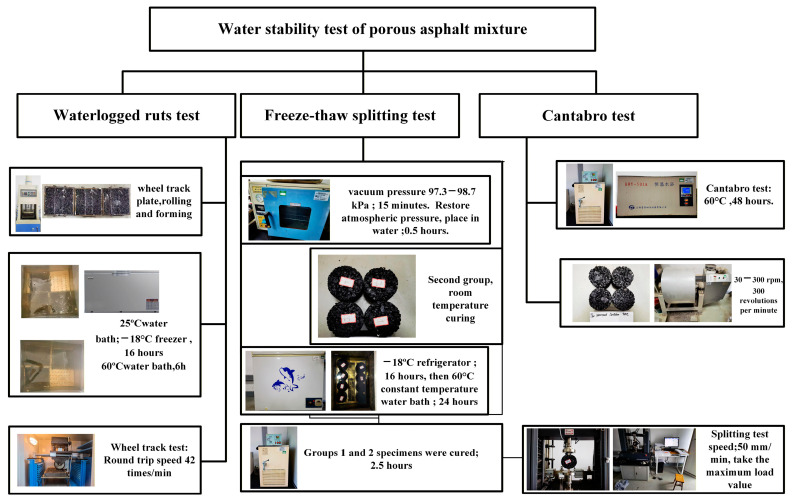
Diagram of testing of graphene-modified permeable asphalt mixture’s water stability performance.

This study employed graphene-modified permeable asphalt mixtures subjected to both short-term and long-term thermo-oxidative ageing to investigate their water stability performance during prolonged service. The freeze–thaw splitting, water immersion Cantabro, and standard Cantabro tests evaluated the water stability under single-factor conditions. These experimental methods were employed to obtain the freeze–thaw splitting tensile strength ratio, mass loss rate, and water immersion mass loss rate, respectively,, respectively Concurrently, a water-soaked rutting test was employed to determine dynamic stability (DS), residual dynamic stability, and relative deformation after 60 min. These metrics analysed the water stability under multi-factor coupled effects. The specific experimental workflow is illustrated in Figure 3.

#### 2.4.1. Thermal Oxygen Ageing Test

In compliance with ‘the Test Procedures for Asphalt and Asphalt Mixtures for Highway Engineering (JTG E20-2011)’ [36] (hereinafter referred to as the ‘Highway Regulations’), short-term and long-term thermal oxidative ageing tests were conducted on graphene-composite-modified permeable asphalt mixtures. The effects of thermal oxidative ageing on the mixtures’ water stability were evaluated using experimental indicators derived from freeze–thaw splitting tests, immersion Cantabro tests, standard Cantabro tests, and multi-factor coupled water immersion rutting tests performed before and after ageing. The short-term test simulated the transient oxidative reactions that occur at elevated temperatures during the production and initial paving stages of asphalt mixtures [37]. During the experiment, the loose permeable asphalt mixture was heated for 4 h in a forced-ventilation drying oven at 135 °C and turned over once every hour. The long-term treatment simulated the gradual deterioration of permeable asphalt mixtures over decades of post-paving service as they are exposed to natural environmental conditions, including solar radiation and atmospheric oxygen exposure [38]. The mixture subjected to thermal-oxygen-accelerated ageing in the experiment was placed in a forced-ventilation drying oven at 85 °C for 120 h, after which it was removed and allowed to cool naturally for at least 16 h to reach ambient temperature.

#### 2.4.2. Freeze–ThawFreeze-Thaw Splitting Test

To investigate the water stability of graphene-modified permeable asphalt mixtures under the sole influence of freeze–thaw cycles, freeze–thaw splitting tests were employed. The max splitting strength values of the specimens were measured before and after freeze–thaw cycles, and the TSR index was calculated accordingly. In accordance with ‘Highway Regulations’, specimens were saturated for 15 min under 97.3 kPa vacuum and then removed and sealed in 10 mL plastic water bags. These were placed in a −18 °C freezer for 16 h, followed by a 24 h incubation in a 60 °C constant-temperature water bath. Finally, after a 2 h period in a 25 °C water bath, splitting tests were conducted to calculate the freeze–thaw splitting tensile strength ratio (TSR).

#### 2.4.3. Cantabro Test

To investigate the water stability of the graphene-modified permeable asphalt mixtures under single-factor dry loading and water-saturated hydrothermal conditions, mass loss rates were measured using standard Cantabro and water-immersed Cantabro tests, respectively [39]. For the standard test, Marshall specimens were cured in water at a constant temperature of 25 °C for 20 h and then dried and taken out for use. For the immersion test, the Marshall samples were cured in water at a constant temperature of 60 °C for 48 h, then cured in water at room temperature for 24 h, and taken out, and the surface moisture was wiped dry for use in the experiment. The cured specimens were then placed in a Los Angeles abrasion tester and rotated at 30–33 r/min for 300 revolutions. The formula for calculating the mass loss rate is as follows.

(6) $$\Delta S=\frac{m_{0}-m_{1}}{m_{0}}$$

where m_0_ and m_1_ denote the masses of the graphene-modified permeable asphalt mixture before and after the Cantabro test, respectively.

#### 2.4.4. Multi-Factor Coupled Water Immersion Rutting Tests

The climate of southern China, characterised by heavy rainfall and flooding in summer and cold, wet winters [40], combined with the severe overloading of vehicles on expressways, readily leads to pavement deterioration. A qualitative evaluation system for the water stability of graphene-modified permeable asphalt mixtures under multi-factor coupled effects was established through water immersion rutting tests, which incorporated three key factors: traffic load, temperature, and ageing degree.

In the water immersion rutting test, wheel loads of 0.7, 0.8, and 0.9 MPa were applied to simulate normal conditions, 50% overload, and 100% overload of permeable asphalt pavements, respectively. Unaged, thermally–oxidativelythermally and oxidatively short-term-aged, and thermally–oxidativelythermally and oxidatively long-term-aged permeable asphalt mixture rutting specimens—roll-compacted—were placed in a 60 °C water bath for 6 h prior to high-temperature water immersion rutting tests. After formation, the specimens were placed in a 20 °C water bath for 2 h, followed by placement in a −18 °C freezer for 16 h. The cured specimens were subjected to freezing water immersion rutting tests using a fully automated asphalt mixture rutting tester, which simulated changes in water stability under coupled water and load effects in both high- and low-temperature extreme conditions.

Through water immersion rutting tests, the DS (dynamic stability), residual dynamic stability ratio, and 60 min relative deformation indices were obtained. A comprehensive analysis was conducted on the variation patterns of water stability in the graphene-modified water-permeable asphalt mixtures under different traffic loads and temperature conditions across three scenarios: non-aged, short-term thermally oxidised, and long-term thermally oxidised. Higher DS values, higher residual dynamic stability ratios, and lower 60 min relative deformations indicated better water stability. DS and the residual dynamic stability ratio are presented in Equations (7) and (8), respectively, which are derived from the ‘Highway Regulations’.

(7) $$\mathrm{DS}=\frac{(t_{60}-t_{45})\times N}{d_{60}-d_{45}}\times C_{1}\times C_{2}$$

In the formula, d_1_ and d_2_ correspond to the deformation values at 45 min (t_1_) and 60 min (t_2_), respectively; C_1_ and C_2_ are the rutting test correction factors, taken as 1.0; and N is the rutting wheel reciprocating speed, set at 42 cycles per millimetre.

(8) $$\mathrm{Residual}\;\mathrm{dynamic}\;\mathrm{stability}\;\mathrm{ratio}\;=\frac{DS_{1}}{DS_{0}}$$

In the formula, DS_0_ and DS_1_ denote the dynamic stability of the graphene-modified permeable asphalt mixture under standard conditions and after immersion testing, respectively.

#### 2.4.5. Dynamic Shear Rheometer (DSR) Test

To verify the formation of a network structure by SBS in the graphene-composite-modified asphalt, rheological analysis was conducted through DSR experiments, considering frequency and the energy storage modulus (G′). The experimental temperature was 64 °C, the strain was 1%, and the scanning frequency range was 0.01–100 rad/s, with the key low-scanning-frequency range being 0.01–0.1 rad/s. The TA HR10 model was used as the experimental instrument.

#### 2.4.6. Raman Mapping Test

The dispersion uniformity of graphene in the asphalt mixtures was analysed through its Raman spectral characteristic peak (G). Raman mapping was used to scan the 20 ×* 20-micron area, and the Horiba HR Evolution model was used as the experimental instrument.

#### 2.4.7. Energy Dispersive Spectrometer (EDS) Testing

To investigate the microstructural characteristics of water stability in the graphene-modified permeable asphalt mixtures in different ageing stages and the damage mechanisms during ageing, EDS surface scanning experiments were conducted to analyse the elemental composition and distribution within the mixtures. The Gemini SEM300 EDS spectrometer model produced by Carl Zeiss of Germany was adopted.

## 3. Results and Discussion

### 3.1. Water Stability Under Freeze–ThawFreeze-Thaw Action

**Figure 4:**
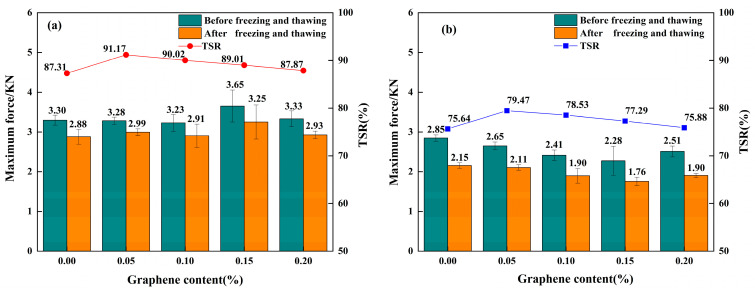
Average maximum force of permeable asphalt mixtures under freeze–thaw action in different ageing states and TSR: (**a**) Average maximum force and TSR of unaged permeable asphalt mixtures. (**b**) Average maximum force and TSR of thermally and oxidatively aged permeable asphalt mixtures.

Through freeze–thaw splitting tests, the TSR index was obtained. A higher TSR value indicates better water stability of graphene-modified permeable asphalt mixtures under the single factor of freeze–thaw cycling. The test data for mixtures in the unaged and thermally–oxidativelythermally and oxidatively aged states are presented in Figure 4a and Figure 4b, respectively. The freeze–thaw splitting test data in the figures are derived from four independent parallel experiments (*n* = 4), with error bars indicating standard deviations.

As shown in Figure 4a, the maximum TSR value of the unaged graphene-composite-modified permeable asphalt mixture with a 0.05% graphene content reached 91.17%, representing a 4.5% increase compared to the TSR value of 87.31% of the unaged SBS-modified mixture. As the graphene content increased, the TSR value decreased yet remained higher than that of the SBS-modified permeable asphalt mixture without graphene addition.

As presented in Figure 4b, the TSR values of thermally–oxidativelythermally and oxidatively aged graphene-modified permeable asphalt mixtures generally exhibited an initial increase followed by a decline. The mixture with 0.05% graphene content exhibited the highest TSR value of 79.47%, representing a 5.1% increase compared with the TSR value of 75.64% for the thermally–oxidativelythermally and oxidatively aged SBS-modified permeable asphalt mixture. As the graphene content further increased, the TSR values of the graphene-modified mixtures gradually decreased. Nevertheless, they remained consistently higher than those of the thermally–oxidativelythermally and oxidatively long-term-aged SBS-modified permeable asphalt mixture. This indicates that graphene incorporation enhances the TSR value of SBS-modified mixtures. By improving physicochemical properties, graphene enhances water stability under freeze–thaw cycling.

Analysis of Figure 4a,b reveals that the TSR values of thermally–oxidativelythermally and oxidatively aged graphene-modified permeable asphalt mixtures with graphene contents of 0%, 0.05%, 0.1%, 0.15%, and 0.2% were reduced by 15.42%, 13.33%, 12.93%, 13.12%, and 13.64%, respectively. The reduction in TSR values for graphene-modified mixtures was consistently lower than that for SBS-modified mixtures. This indicates that graphene incorporation mitigates the degradation rate of water stability in thermally–oxidativelythermally and oxidatively aged permeable asphalt mixtures under freeze–thaw cycles, thereby improving their long-term water stability.

### 3.2. Water Stability Under Load

**Figure 5:**
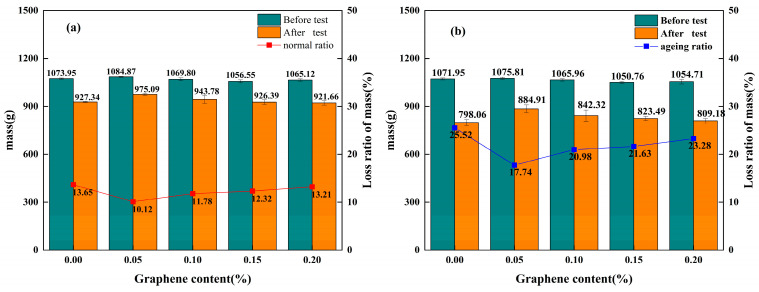
Average mass and mass loss rate of permeable asphalt mixtures before and after testing under different ageing conditions: (**a**) Average mass and mass loss rate of unaged permeable asphalt mixtures before and after testing. (**b**) Average mass and mass loss rate of thermo-oxidatively aged permeable asphalt mixtures before and after testing.

Through standard Cantabro tests, the mass loss rates were obtained. A lower rate indicates better water stability of graphene-modified permeable asphalt mixtures under single-factor loading conditions. The standard Cantabro test data for unaged and thermally–oxidativelythermally and oxidatively aged permeable asphalt mixtures are presented in Figure 5a and Figure 5b, respectively. The data in the figures are derived from four independent parallel experiments (*n* = 4), with error bars indicating standard deviations.

As shown in Figure 5a,b, the mass loss rates of both unaged and aged graphene-modified permeable asphalt mixtures under water-load conditions exhibited a trend of an initial decrease followed by an increase with increasing graphene content. At a 0.05% graphene content, both unaged and aged mixtures exhibited the lowest mass loss rates of 10.12% and 17.74%, respectively, corresponding to the highest water stability under water-load conditions. Conversely, at a 0% graphene content, both unaged and aged mixtures recorded the highest mass loss rates of 13.65% and 25.52%, respectively. This data indicates that at a 0.05% content, both graphene-modified permeable asphalt mixtures exhibited a 25.64% improvement in water stability under load compared to SBS-modified mixtures. Additionally, aged mixtures demonstrated a 23.04% enhancement in water stability. Simultaneously, comparison of the mass loss rates between unaged and thermally–oxidativelythermally and oxidatively aged graphene-composite-modified permeable asphalt mixtures reveals that the overall trend for the graphene-composite-modified mixture—which was prepared by adding graphene to SBS-modified asphalt—exhibited a relatively gentle curve. This mixture demonstrated better water stability under load, with smaller fluctuations.

As shown in Figure 5a,b, the mass after the standard Cantabro test first increased and then decreased with the graphene content. At the same graphene content, the post-test masses of the thermally and oxidatively aged graphene-modified permeable asphalt mixtures were consistently lower than those of unaged samples. This indicates that thermal oxidative ageing exacerbates the susceptibility of graphene-modified permeable asphalt mixtures to water damage under loading conditions. The mass loss rates of thermally–oxidativelythermally and oxidatively aged mixtures at graphene dosages of 0%, 0.05%, 0.1%, 0.15%, and 0.2% were increased by 84.61%, 75.31%, 78.09%, 75.56%, and 76.23%, respectively. This indicates that graphene incorporation mitigates the decline in water stability in permeable asphalt mixtures under loading conditions after thermal oxidative ageing. As a two-dimensional layered material, graphene stacks layer by layer within the asphalt matrix; this impedes the thermal oxidative ageing reaction, thereby enhancing the water stability of graphene-composite-modified permeable asphalt mixtures during long-term service.

### 3.3. Water Stability Under Hydrothermal Conditions

**Figure 6:**
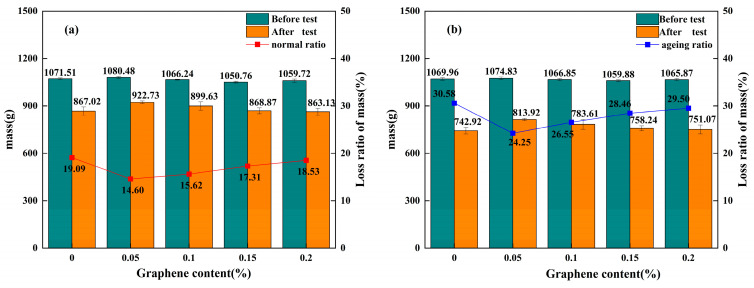
Average mass before and after testing and water immersion mass loss rate of permeable asphalt mixtures under different ageing conditions: (**a**) Average mass before and after testing and water immersion mass loss rate of unaged permeable asphalt mixture. (**b**) Average mass before and after testing, and water immersion mass loss rate of permeable asphalt mixture subjected to prolonged thermal–oxidativethermal oxidative ageing.

The immersed Cantabro test yielded immersed mass loss rate metrics. A lower rate indicates superior water stability of graphene-modified permeable asphalt mixtures under hydrothermal single-factor action. Data from the immersed Cantabro tests for unaged and thermally aged mixtures are shown in Figure 6a and Figure 6b, respectively. The data are derived from four independent parallel experiments (*n* = 4), with error bars indicating standard deviations.

As shown in Figure 6a, when the graphene content increased from 0% to 0.05%, the water immersion mass loss rate of the unaged graphene-composite-modified permeable asphalt mixture decreased from 19.09% to 14.60%, a reduction of 23.52%. At graphene content levels of 0.1%, 0.15%, and 0.2%, the water immersion mass loss rates were 15.62%, 17.31%, and 18.53%, respectively, all lower than that of the unmodified mixture. This indicates that graphene, when employed as a modifier in SBS permeable asphalt mixtures, enhances hydrothermal stability under single-factor water exposure. However, excessive graphene incorporation may induce agglomeration, where the resulting aggregates may not be fully encapsulated by the SBS-modified asphalt. This reduces the adhesion between the asphalt and aggregate, potentially leading to increased water erosion and consequently, lower mass loss rates.

As shown in Figure 6b, when the graphene content increased from 0% to 0.05%, the water immersion mass loss rate of the aged graphene-modified permeable asphalt mixture decreased from 30.58% to 24.25%, representing a reduction of 20.70%. At dosages of 0.1%, 0.15%, and 0.2%, the water immersion mass loss rates were 26.55%, 28.46%, and 29.50%, respectively, all exceeding those of the aged SBS-modified control group. This indicates that graphene incorporation generally enhances the water stability of aged SBS-modified permeable asphalt mixtures. With an increase in graphene content from 0.05% to 0.2%, the water loss rate curve for aged samples exhibits less pronounced steepness compared to the unaged curve. This demonstrates that graphene addition enhances the hydrothermal stability of permeable asphalt mixtures.

As shown Figure 6a,b, the post-test mass exhibited a trend of initially increasing then decreasing with rising graphene content. At identical graphene dosages, the post-test mass of long-term thermal-oxygen-aged graphene-composite-modified mixtures consistently fell below that of non-aged samples. This indicates that permeable asphalt mixtures become more susceptible to water erosion under ageing conditions, thereby diminishing their resistance to water damage. The water immersion mass loss rates of aged mixtures at 0%, 0.05%, 0.1%, 0.15%, and 0.2% graphene contents increased by 60.18%, 66.09%, 69.97%, 64.41%, and 59.2%, respectively. The water loss rate of aged graphene-composite-modified mixtures under hydrothermal action exhibited a greater increase than that of SBS-modified mixtures, indicating that hydrothermal action exacerbates water damage in graphene-modified permeable asphalt mixtures. The flake-like structure of graphene forms microchannels for water molecules during hydrothermal treatment and thermo-oxidative reactions, along which water permeates into the interface between the asphalt and aggregate, diminishing the adhesion between them. Consequently, this reduces the water stability performance of graphene-composite-modified permeable asphalt during ageing.

### 3.4. Water Stability Under Load, Temperature, and Ageing Effects

In the multi-factor coupling experiment, graphene-modified permeable asphalt mixtures—comprising unaged, thermo-oxidatively short-term-aged, and thermo-oxidatively long-term-aged variants—were subjected to freeze–thaw cycles, loading, and hydrothermal stress. The graphene dosage of 0.05% yielded optimal performance in the TSR, mass loss rate, and water immersion mass loss rate metrics. This dosage was adopted for the multi-factor coupled water immersion rutting test.

#### 3.4.1. Water Stability Under Load, High Temperature, and Ageing Effects

**Figure 7:**
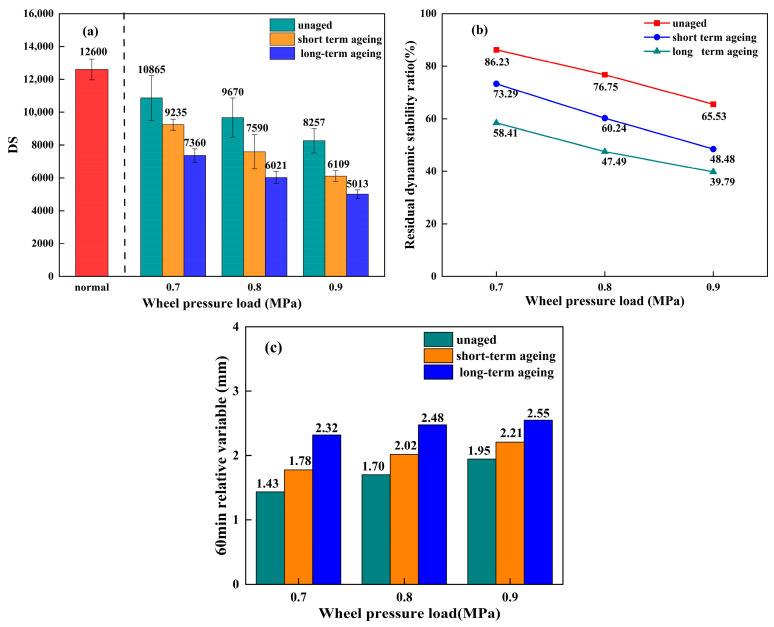
High-temperature water immersion rutting test data for unaged, short-term thermo-oxidatively aged, and long-term thermo-oxidatively aged permeable asphalt mixtures: (**a**) DS. (**b**) Residual dynamic stability ratio. (**c**) Relative deformation after 60 min.

The high-temperature water immersion rutting test indicators—DS, the residual dynamic stability ratio, and relative deformation at 60 min—are, respectively, represented in Figure 7a–c. The data in the figure are derived from three independent parallel experiments (*n* = 3), with error bars indicating standard deviations.

As shown in Figure 7a, the average DS value for the standard rutting test with a wheel load of 0.7 MPa corresponded to a relative deformation of 1.8% after 60 min. As the degree of ageing in the permeable asphalt mixtures intensified, their average DS values exhibited a decreasing trend with increasing wheel load; however, the average DS values for the high-temperature water immersion rutting test remained higher than those in the drainage asphalt specification. This indicates that even under the most unfavourable conditions of a wheel load of 0.9 MPa and prolonged thermal–oxidativethermal oxidative ageing, the mixture still possessed the water damage resistance capability required by the specification at elevated temperatures.

As shown in Figure 7b, the residual dynamic stability ratios of permeable asphalt mixtures modified with graphene composites at different ageing levels all exhibited a continuous decline with increasing wheel load during high-temperature immersion testing. For unaged mixtures, the residual dynamic stability ratio decreased from 86.23% to 65.53% when wheel load increased from 0.7 MPa to 0.9 MPa, representing a 20.7% reduction. For thermally and ozonically short-term-aged mixtures under the same wheel load variation, this ratio decreased from 73.29% to 48.48%, representing a 24.81% reduction. Under identical wheel load variations, the residual dynamic stability of thermally oxygenated, long-term-aged permeable asphalt mixtures decreased from 58.41% to 39.79%, representing a reduction of 18.62%.

As shown in Figure 7c, the 60 min relative deformation values of graphene-modified mixtures with varying degrees of ageing all exhibited an upward trend with increasing wheel load. When this load increased from 0.7 MPa to 0.9 MPa, the relative deformation at 60 min for the unaged, thermo-oxidatively short-term-aged, and thermo-oxidatively long-term-aged graphene-composite-modified permeable asphalt mixtures increased by 18.88%, 13.48%, and 9.91%, respectively. As ageing progressed, the rate of increase in this deformation diminished. This indicates that graphene addition mitigated the decline in water stability following ageing of the permeable asphalt mixtures. This might be because the multilayered sheet-like structure of graphene itself slows down the effect of thermal–oxidativethermal oxidative ageing.

#### 3.4.2. Water Stability Under Load, Low Temperature, and Ageing Effects

**Figure 8:**
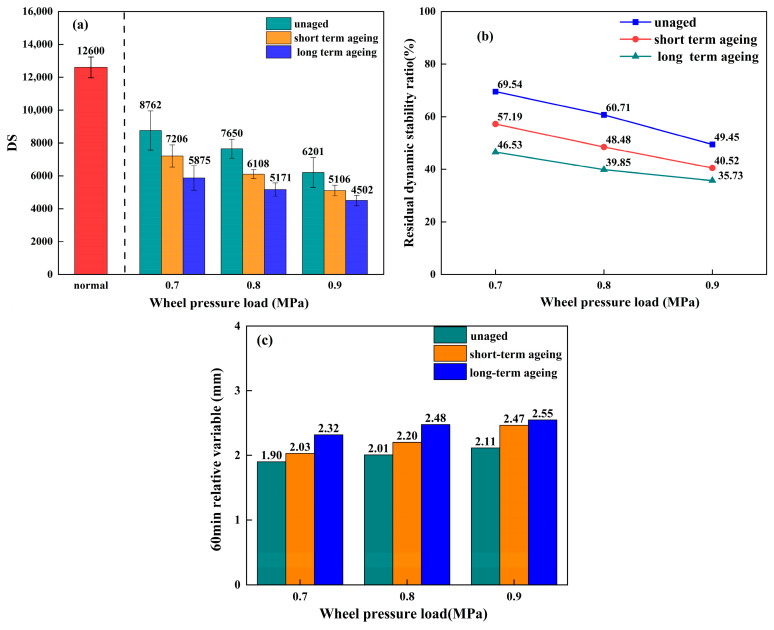
Low-temperature water immersion rutting test data for unaged, thermally and oxidatively short-term-aged, and thermally and oxidatively long-term-aged permeable asphalt mixtures: (**a**) Average DS. (**b**) Residual dynamic stability ratio. (**c**) Relative deformation after 60 min.

Figure 8a, Figure 8b, and Figure 8c, respectively, represent the average DS value, residual dynamic stability ratio, and relative deformation after 60 min for graphene-modified permeable asphalt mixtures subjected to freeze–thaw water rutting tests under different ageing conditions and wheel load pressures. The low-temperature water immersion test data in the figure are derived from three independent parallel experiments (*n* = 3), with error bars indicating standard deviations.

As shown in Figure 8a, the average DS values of graphene-composite-modified permeable asphalt mixtures under various ageing conditions all exhibited a decreasing trend with increasing wheel load during freeze–thaw immersion tests. Moreover, these values consistently exceeded the specifications for porous asphalt. This indicates that even under the most unfavourable conditions—prolonged thermo-oxidative ageing combined with heavy loading at 0.9 MPa—the graphene-composite-modified mixtures maintained satisfactory resistance to water damage at low temperatures.

As shown in Figure 8b, the residual dynamic stability ratio of freeze–thaw-treated permeable asphalt mixtures modified with graphene composites at different ageing levels consistently decreased with increasing wheel load. For unaged mixtures, the residual dynamic stability ratio decreased from 69.54% to 49.45% when wheel load increased from 0.7 MPa to 0.9 MPa, representing a reduction of 28.89%. For thermally and ozonically aged mixtures under identical wheel load variations, this ratio decreased from 57.19% to 40.52%, representing an 11.84% reduction. Under identical wheel load variations, the residual dynamic stability of thermally oxygenated long-term-aged permeable asphalt mixtures decreased from 46.63% to 35.73%, representing a reduction of 23.37%.

Based on the comprehensive analysis of the high-temperature water immersion rutting test and freezing water immersion rutting test indicators for the graphene-modified permeable asphalt mixture, the water stability of this mixture meets practical requirements under the most adverse conditions, including wheel load and ageing. Water stability under high-temperature immersion conditions proves superior to that under freeze–thaw conditions. This may be attributed to graphene’s superior thermal stability. Concurrently, graphene’s unique dense atomic arrangement and hydrophobicity create a physical barrier effect within the asphalt matrix, effectively impeding water penetration and erosion. This maintains the stability of the asphalt–aggregate interface structure while inhibiting moisture-induced degradation processes in graphene-modified permeable asphalt mixtures, ultimately enhancing their water stability.

The multi-factor coupled immersion rutting test simulates the variation patterns of water stability in permeable asphalt mixtures within humid, hot, and rainy regions. The experimental findings demonstrate that graphene incorporation significantly delays the decline in the water stability of these mixtures, thereby providing theoretical justification for their application in heavy-duty traffic and long-life pavement design.

### 3.5. Analysis of Rheological Results

**Figure 9:**
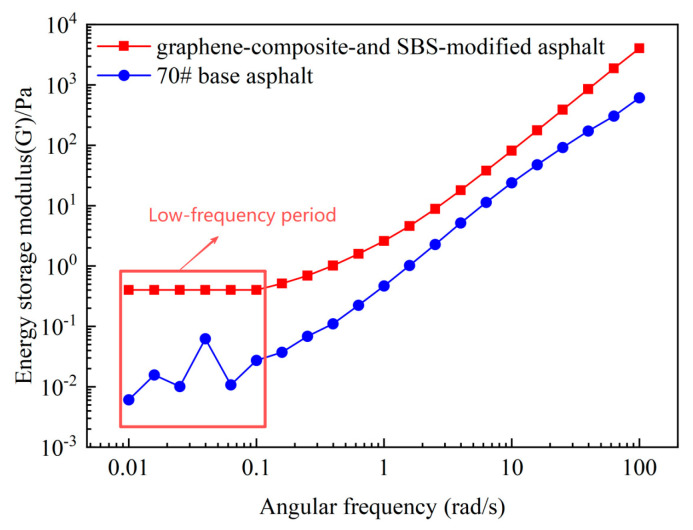
The angular frequency–g′ log–log coordinates graph of the full angular frequency. The graphene content in graphene-composite -and SBS-modified asphalt was 0.05%, and the DSR experimental temperature was 64 °C.

The G′ values of 70# base asphalt and graphene-composite- and SBS-modified asphalt in the angular frequency range of 0.01–100 rad/s in the log–log coordinates graph are shown in Figure 9.

As can be seen from Figure 9, in the full angular frequency range of 0.01–100 rad/s, the G′ of the graphene-composite- and SBS-modified asphalt is higher than that of 70# base asphalt at the same angular frequency. In the low-frequency range of 0.01–0.1 rad/s, the G′ changes in graphene-composite- and SBS-modified asphalt are not significant, and the slope tends to 0, and there is a plateau period. However, the G′ slope of 70# base asphalt within this range varies greatly and does not show the plateau region. The G′ variation phenomenon of graphene-composite- and SBS-modified asphalt indicates that graphene and SBS additives form a three-dimensional network structure in asphalt.

## 4. Microscopic Analysis of Graphene-Modified Permeable Asphalt Mixture

### 4.1. Analysis of the Uniformity of Graphene Dispersion

**Figure 10:**
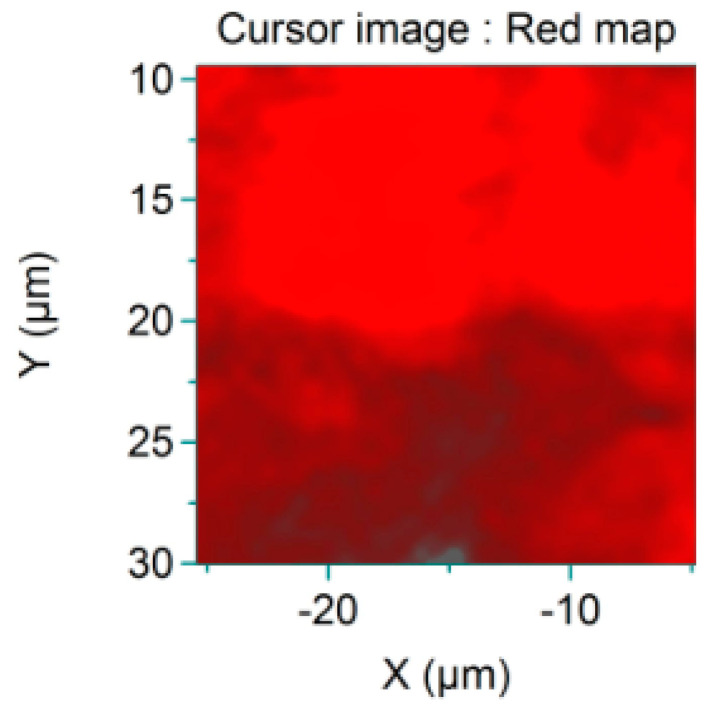
Diagram of graphene dispersion.

Uniform dispersion of the graphene is shown in Figure 10, where red represents the unique Raman peaks. The brighter the red is, the higher the intensity of the peaks is, and, accordingly, the greater the content of graphene is. A darker red colour indicates a lower peak intensity and lower graphene content.

As can be seen from Figure 10, the red area in the figure is widely distributed throughout the entire region, which indicates that the graphene had good uniformity in dispersion in the modified asphalt. This indirectly enhanced the water stability of the graphene-composite-modified permeable asphalt mixture.

### 4.2. Distribution of Chemical Elements in Graphene-Modified Permeable Asphalt Mixtures at the Microscopic Level

In this study, we selected oxygen, carbon, sulphursulfur, silicon, and calcium for surface element distribution research and analysed the element laws that affected the water stability performance of graphene-composite-modified mixtures under long-term service. The fundamental mechanism of permeable asphalt mixture ageing is oxidative reaction [41], and the degree of oxidation in different ageing states is characterised by oxygen distribution. Graphene is a material comprising a single layer of carbon atoms arranged in a honeycomb lattice structure [42]. Basalt aggregates are mainly composed of SiO_2_ and CaO, with the content of the former higher than that of the latter [43]. The aggregates in this study were characterised by Si and Ca elements.

**Figure 11:**
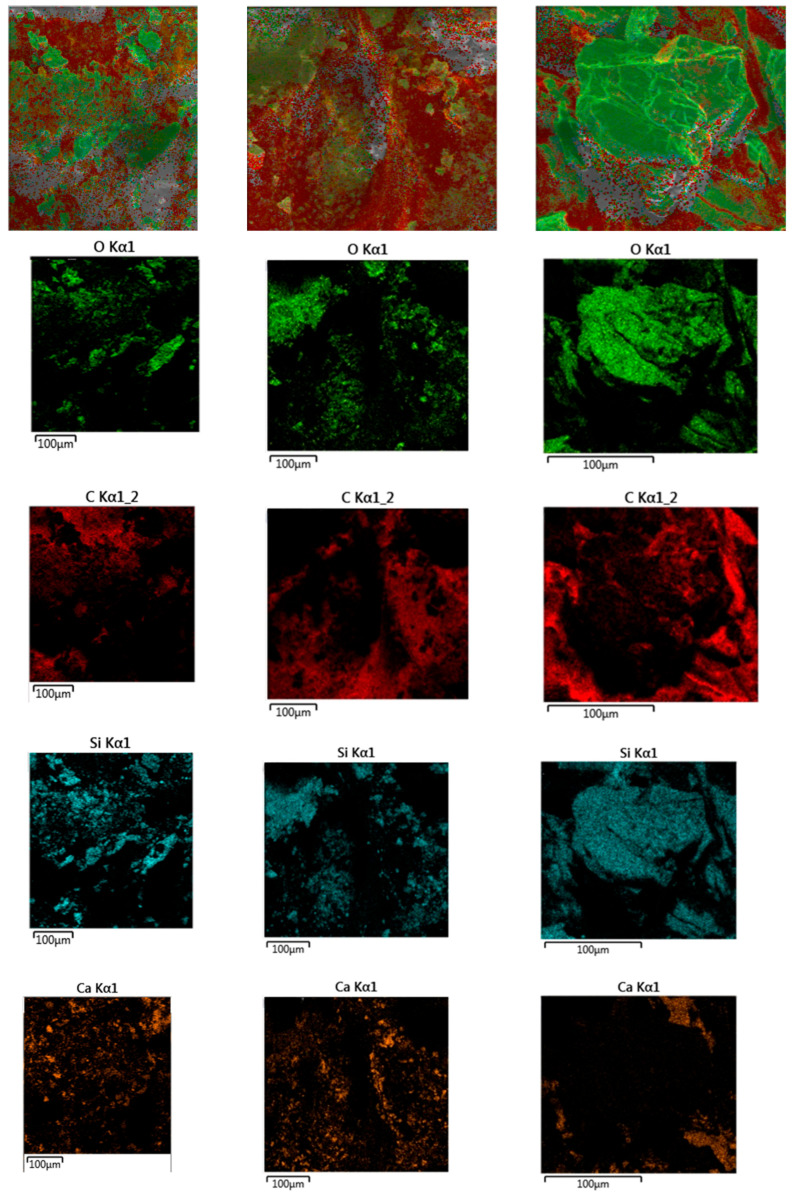

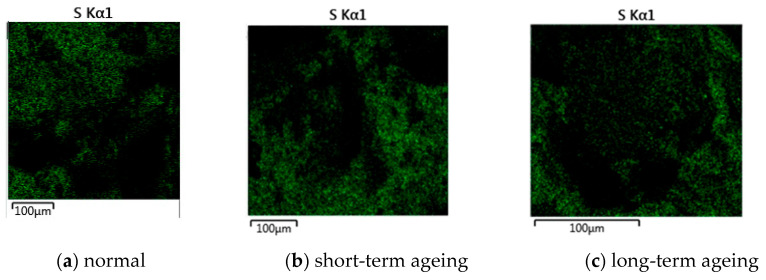
Elemental distribution in permeable asphalt mixtures at different degrees of ageing: (**a**) Element distribution in the unaged state. (**b**) Element distribution after short-term thermo-oxygen ageing. (**c**) Element distribution after long-term thermo-oxygen ageing.

The elemental distribution maps of graphene-modified permeable asphalt mixtures under non-aged, short-term ageing, and long-term ageing conditions in the water-soaked rutting test are shown in Figure 11a, Figure 11b, and Figure 11c, respectively. The colour intensity in the elemental distribution maps correlates positively with the relative concentration of elements: darker colours indicate higher brightness in the distribution map and thus greater relative elemental content.

As shown in Figure 11, oxygen elements exhibit increasingly deeper colour intensity and higher concentration as the ageing degree of the graphene-modified permeable asphalt mixtures intensifies. The carbon element maintains a relatively high overall concentration across all ageing stages of the mixtures. Analysis of combined silicon and calcium distribution maps reveals that, across all ageing stages, the concentration of silicon consistently surpasses that of calcium within the mixtures. This is primarily attributable to the use of basalt for both coarse and fine aggregates in this study, which exhibits a higher CaO content relative to its SiO_2_ content. The ageing process of the mixtures did not significantly affect the silicon or calcium content.

**Figure 12:**
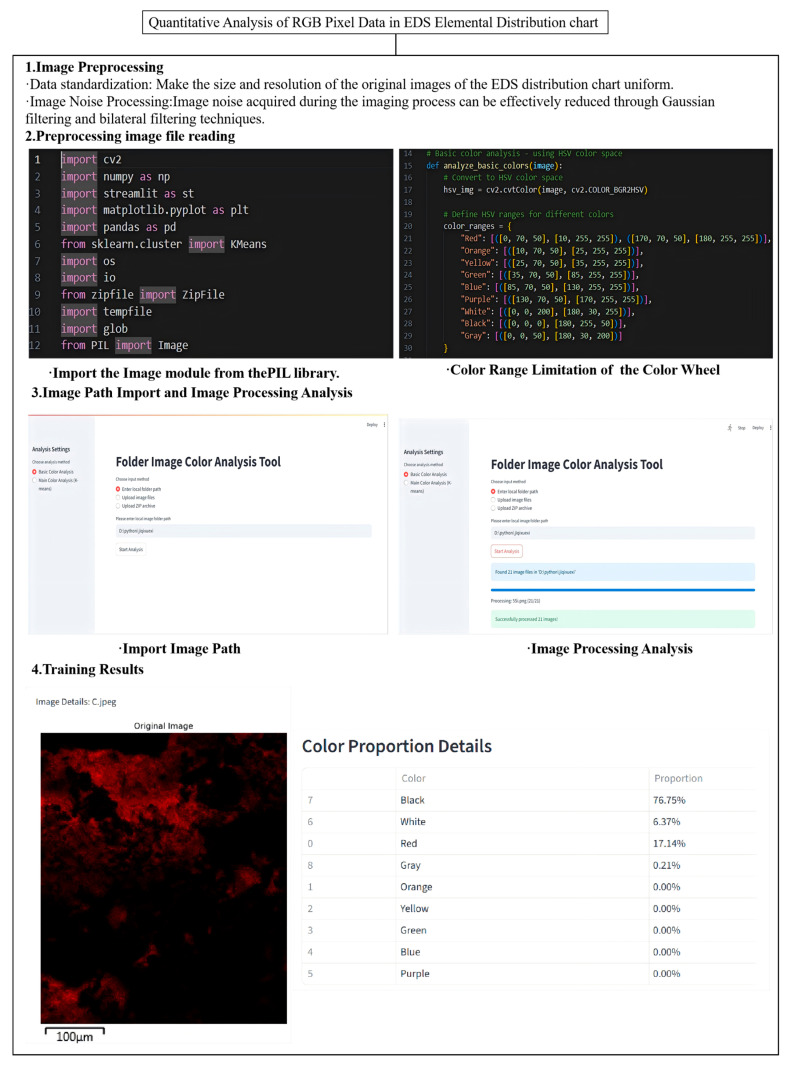
Analysis diagram of the RGB algorithm for image pixel processing.

**Figure 13:**
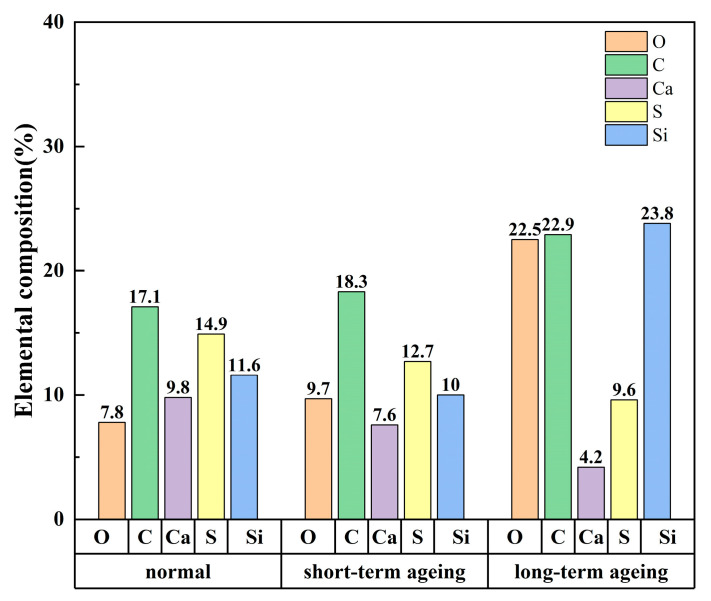
EDS elemental quantitative proportion chart.

To quantitatively characterise the element concentration in the EDS surface element distribution map, we adopted the RGB algorithm for image pixels. Essentially, this is a colour algorithm based on the principle of additive colour mixing. The representative colour of each element in the distribution diagram can be formed by the three primary colours—R (red), G (green), and B (blue)—at different pixel ratios. The specific operation of the RGB algorithm is shown in Figure 12. The RGB algorithm could innovatively quantitatively characterise the proportion of each element in the figure, thereby determining the element content of graphene-composite-modified permeable asphalt mixtures under different degrees of ageing, shown in Figure 13.

As can be seen from Figure 13, during the process from non-ageing to short-term ageing of graphene-composite-modified permeable asphalt, the oxygen content increased from 7.8% to 9.7%, but the growth rate was not significant. As the thermo-oxidative ageing reaction intensified, the oxygen content of the long-term-aged mixture rose to 22.5%, representing a 2.9-fold increase compared to that of the unaged state. This aligns with the broader trend of a declining TSR and rising quality loss rates. The ageing of graphene-composite-modified mixtures exhibited a positive correlation with changes in oxygen content. The carbon content in unaged, short-term-aged, and long-term-aged graphene-composite-modified permeable asphalt mixtures was 17.1%, 18.3%, and 22.9%, respectively; the sulphur content was 14.9%, 12.7%, and 9.6%, respectively. A three-dimensional network structure formed within the SBS-modified bitumen, and graphene enhances enhanced the compactness of this three-dimensional network through physical adsorption. The sulphur content decreased as thermal oxygen ageing reactions intensified. Graphene, through physical action and hydrophobicity, worked together with the SBS-modified asphalt to effectively prevent water from penetrating into the permeable asphalt mixture, thereby enhancing the water stability of the mixture in the unaged state. During thermal–oxidativethermal oxidative ageing, sulphur-containing functional groups in bitumen—sulphones, sulphides, and mercaptans—underwent oxidation reactions to produce SO_2_ or SO_3_ gas. This gas, when heated, accelerated its escape, thereby reducing the sulphur content.

## 5. Conclusions

In this study, we comprehensively investigated the water stability performance of graphene-composite-modified permeable asphalt mixtures under various service factors. The principal conclusions are summarised as follows:

(1) An optimal graphene content of 0.05% enhanced water stability under various conditions. It yielded the highest performance, with unaged mixtures achieving a TSR of 91.17%, mass loss rate of 10.12%, and immersion mass loss rate of 14.60%. After long-term thermo-oxidative ageing, these values changed to 79.47%, 17.74%, and 24.25%, respectively.

(2) Graphene delays ageing effects; it reduces water stability across freeze–thaw cycles and loading conditions, attributable to its two-dimensional layered structure, which hinders oxidative ageing. However, under hydrothermal conditions, graphene exacerbates the immersion mass loss due to its layered nature accelerating moisture intrusion.

(3) At high temperatures, graphene-reinforced mixtures exhibit superior rutting resistance compared to those under freezing conditions, owing to graphene’s inherent thermal stability. Even under harsh hydrothermal ageing at 0.9 MPa, water stability remains satisfactory due to graphene’s hydrophobic properties.

(4) Microstructurally, graphene is uniformly dispersed in graphene-composite- and SBS-modified asphalt and presents a three-dimensional network structure in rheology. Graphene and SBS enhance the water stability of permeable asphalt mixtures. With the accelerated ageing of these mixtures, the oxygen content gradually increases, and sulphursulfur compounds react to form SO_2_ or SO_3_.

In summary, an optimised 0.05% graphene content effectively enhances the water stability of permeable asphalt pavements under complex environmental conditions and extends their service life. The limitations of this study lie in the fact that the experiments were primarily conducted under controlled laboratory conditions, failing to fully account for scenarios encountered in actual engineering practice. Subsequent research may investigate the variation patterns of water stability under coupled thermal oxygen and ultraviolet ageing conditions.

## Figures and Tables

**Table 1:** Main technical indications of multilayer graphene [31].

Purity /%	Layer Size	Number of Layers	Oxygen Content /%	Sulphur Content /%	Specific Surface Area /m^2^/g
>95	5–50	6–10	0.5	0.5	100–300

## Data Availability

The original contributions presented in this study are included in the article. Further inquiries can be directed to the corresponding author.
